# Co-Designing a Clinician-Centered Video Gait Assessment System for Cerebral Palsy Based on the Framework for Co-Design of Clinical Practice Tools: Iterative Development and Formative Evaluation Study

**DOI:** 10.2196/92823

**Published:** 2026-08-21

**Authors:** Xi Gao, Xingye Cheng, Jiming Sun, Wenqi Liang, Stacey Reading, Yanxin Zhang

**Affiliations:** 1 School of Exercise, Sport and Rehabilitation Sciences, Faculty of Science, University of Auckland, Bldg 907, 368 Khyber Pass, Auckland, 1023, New Zealand, 64 9 3737599 ext 86859; 2 Qingdao Eber Hospital, Qingdao, Shandong, China

**Keywords:** co-design, gait analysis, cerebral palsy, decision-support system, rehabilitation, human factors

## Abstract

**Background:**

Clinical gait assessment is essential for monitoring functional progress in children with cerebral palsy (CP); however, traditional visual observation remains inherently subjective and labor-intensive. Although AI-supported video gait assessment may provide more objective and automated outputs, many tools remain difficult to integrate into routine clinical workflows.

**Objective:**

This study aimed to iteratively develop and evaluate a clinician-centered, automated gait analysis system for children with CP by using a structured co-design process, ensuring the tool effectively supports clinical decision-making and integrates into routine practice.

**Methods:**

This study adopted the Framework for Co-design of Clinical Practice Tools (FRESCO) to guide a 5-step iterative development process. A multidisciplinary advisory group, comprising rehabilitation physicians, therapists, biomechanics experts, and human factors engineers, collaborated throughout the study. The process involved the following: (1) identifying baseline clinical needs and initial system requirements through advisory group input and review of existing gait-analysis systems; (2) developing an initial system prototype based on these requirements; (3) conducting think-aloud evaluations and System Usability Scale (SUS) assessments with 19 rehabilitation professionals to identify usability barriers, additional user requirements, and prototype refinement needs; (4) testing workflow integration and safety in clinical simulations with 12 rehabilitation professionals; and (5) finalizing prototype specifications, workflow procedures, and implementation guidelines through a consensus workshop.

**Results:**

By targeting workflow alignment, interpretability, and the delivery of useful system outputs, the iterative co-design process supported progressive prototype refinement. The baseline needs-identification phase generated initial system requirements across system content, workflow integration, and clinical usability support, which informed the first prototype. The think-aloud phase demonstrated good perceived usability (SUS score: mean 75.8, SD 7.5) and identified additional refinement needs related to functional expansion, report interpretability, and workflow compatibility. In the clinical simulation phase, the prototype was perceived as stable and safe for use in routine clinical settings, with no safety incidents observed, while also revealing implementation needs related to video capture and output interpretation. This work culminated in 2 key outputs: a refined prototype ready for large-scale clinical testing and a comprehensive implementation guideline. Conceptually, the study articulated two data-informed design principles: (1) supporting workflow-compatible implementation by minimizing technical and operational barriers, and (2) prioritizing clinical autonomy and actionability.

**Conclusions:**

FRESCO provided a structured approach for narrowing the gap between technical feasibility and clinical utility in the development of a clinician-centered video gait assessment system. By iteratively involving a multidisciplinary team, we transformed a video-based analysis tool into a stable, interpretable, and workflow-compatible decision-support prototype. Broadly, the identified design principles may provide practical reference points for developing clinician-centered digital health tools intended to support clinical decision-making in routine practice.

## Introduction

Cerebral palsy (CP) is a permanent and irreversible neurological disorder caused by early brain injury, with a prevalence exceeding 2.0 per 1000 live births [1,2]. Although approximately 75% of children with CP are ambulatory, many present with heterogeneous and progressive gait abnormalities that can adversely affect walking efficiency, balance, and long-term musculoskeletal health [3,4]. Consequently, the timely identification and longitudinal monitoring of gait abnormalities are critical components of effective CP rehabilitation.

In routine clinical practice, gait analysis is commonly based on visual evaluation by clinicians, either through real-time clinical observation or retrospective review of a recorded video. However, such analyses are inherently subjective and difficult to standardize across assessors and settings [5]. Although 3D marker-based gait analysis is considered the reference standard for objective and quantitative biomechanical assessment, its reliance on specialized laboratories, extensive marker placement, trained personnel, and costly equipment limits its feasibility for routine, time-efficient, and longitudinal use in everyday clinical practice [6,7]. More recently, advances in computer vision and AI have further driven the emergence of automated, video-based gait analysis (VGA) systems. These systems enable the extraction of spatiotemporal parameters and clinically relevant gait features directly from conventional RGB video recordings, without the need for complex laboratory infrastructure [8]. From a digital health perspective, these developments enable gait analysis to be more readily integrated into routine clinical workflows, supporting scalable deployment and more consistent decision-making across diverse care settings [9].

Despite these advantages, these systems have seldom been adopted into routine clinical practice, and most remain confined to research use [10]. Previous reviews and clinician-focused studies suggest that this translational gap arises from a set of interrelated usability and implementation barriers [11,12]. First, poor workflow alignment and operational complexity are frequently reported. Many systems (eg, OpenCap) require controlled setup, calibration, or extra prolonged data processing that increase procedural burden and conflict with time-constrained clinical environments [13-15]. Second, automated gait analysis systems often impose a substantial cognitive burden on clinicians due to limited interpretability of their outputs. Results are frequently presented as raw kinematic waveforms or numerical tables, requiring specialist expertise to translate high-dimensional data into clinically meaningful judgments within routine clinical workflows [11,16]. Third, misalignment between system outputs and clinical decision-making needs remains a challenge. Many automated gait analysis systems prioritize tasks that are methodologically convenient and research-relevant, such as predicting Gross Motor Function Classification System levels or discriminating children with CP from healthy controls [17,18]. While clinically meaningful, these outputs may offer limited support for treatment planning, compared with analyses that explicitly characterize specific gait deviations and their severity to inform intervention decisions [19]. Therefore, addressing these challenges requires the adoption of systematic design and implementation strategies that prioritize clinical usability, workflow integration, and decision-relevant outputs, in order to support meaningful translation of VGA systems into routine clinical practice.

User-centered design (UCD) is among the most well-established approaches for supporting the usability of clinical tools [20]. Seeking to enhance the usability of products and systems through a focus on user needs and perspectives, UCD methods are distinguished by their systematic and typically iterative approach to optimizing design through the consideration of contexts of use, usability goals, user characteristics, environments, tasks, and workflows [21,22]. In contrast to traditional top-down waterfall approaches, which often defer testing and feedback to late stages of development, UCD enables earlier identification and resolution of fundamental design issues that may otherwise be difficult to address post hoc [21]. This aligns with broader human-centered and sociotechnical perspectives in digital health, which emphasize that successful clinical technologies require attention not only to device functionality, but also to users, workflows, implementation settings, and the social organization of care [23,24]. However, despite the recognized value of UCD approaches in clinical tool development, their application within VGA systems has often been informal, fragmented, or limited to late-stage usability testing [25-28], rather than implementation as a structured and continuous design framework throughout system development. Moreover, there remains limited practical guidance on how UCD principles can be systematically operationalized in the development of VGA systems, particularly those intended to support complex clinical reasoning and decision-making.

Therefore, the primary aim of this study was to examine how a structured co-design process can support the development of a clinician-centered decision-support VGA system for children with CP. The Framework for Co-design of Clinical Practice Tools (FRESCO) [22], a structured framework that integrates UCD methods and co-design principles for prototype clinical tool development, was used to guide the whole process. To our knowledge, this is the first reported application of FRESCO to the development of a VGA decision-support system for CP rehabilitation. The contribution of this study lies not simply in developing another automated gait analysis prototype, but also in providing practical insights into eliciting clinical and workflow requirements and iteratively refining prototype usability, interpretability, and workflow integration through multidisciplinary stakeholder involvement.

## Methods

### Ethical Considerations

This study was conducted in accordance with relevant laws and institutional guidelines. Ethics approval was granted by the Auckland Health Research Ethics Committee (AHREC; reference number AH26160) and the institutional ethics committee of Shanghai Eber Hospital (20231127).

All participating professionals provided informed consent before taking part in the co-design process. For the use of children’s clinical gait video data, informed consent was obtained from their legal guardians under the approved ethics protocols. All clinical video and assessment data used in this study were deidentified before analysis. Participant information, interview transcripts, observation notes, questionnaire responses, and system-generated outputs were stored securely and reported only in deidentified or aggregated form. No identifiable participant information is presented in this manuscript. No financial compensation was provided to study participants.

### Overview

**Figure 1.**
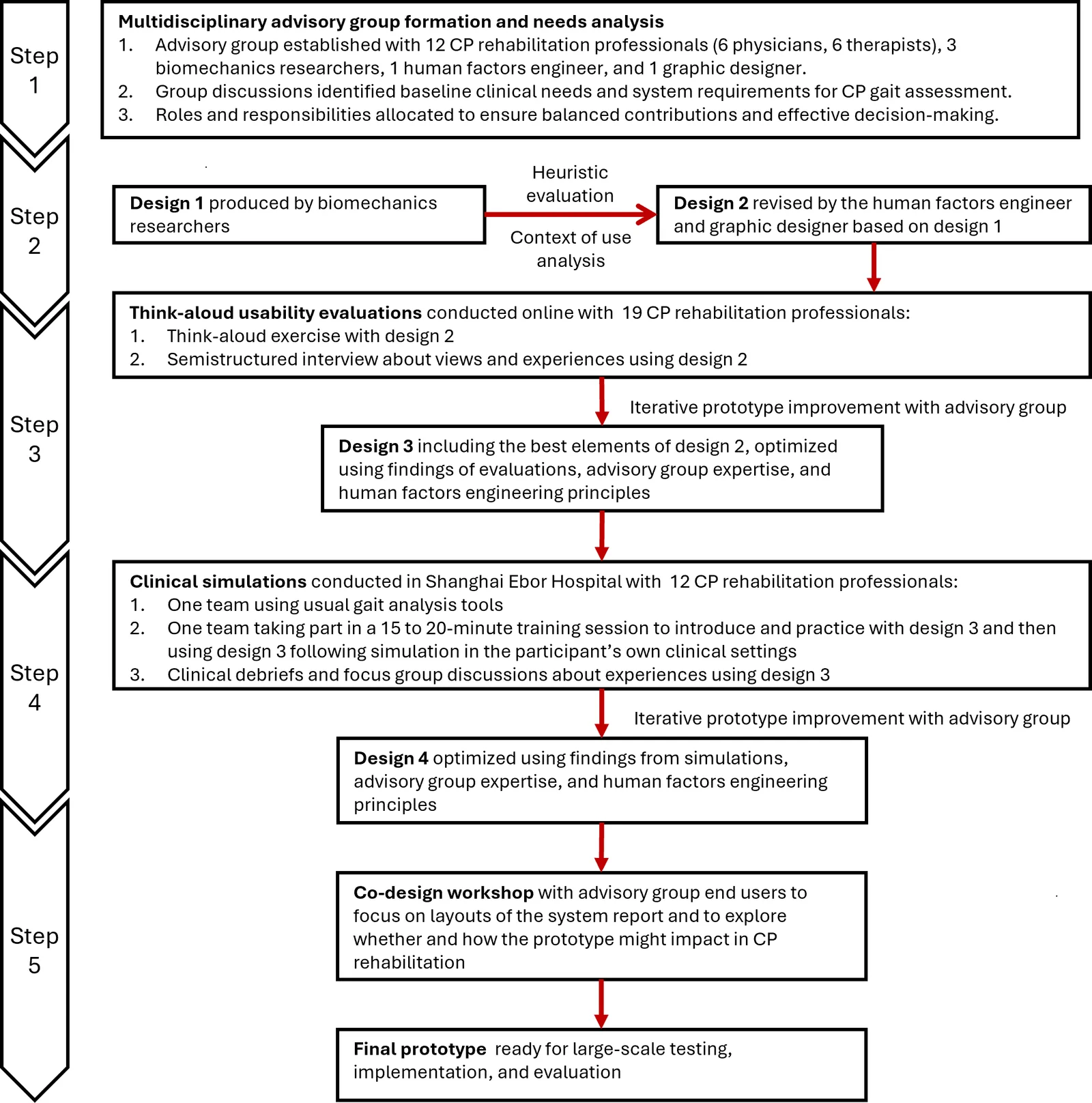
Operational workflow of the Framework for Co-design of Clinical Practice Tools–guided co-design process, showing activities, participants, and iterative refinements. CP: cerebral palsy.

This study followed FRESCO [22] to iteratively develop and formatively evaluate a clinically oriented VGA decision-support prototype for children with CP. Operationalization of FRESCO in this study comprised five steps, each ending with concrete deliverables that informed the next cycle: (1) establishing a multidisciplinary advisory group to identify clinical needs and define initial functional specifications; (2) developing an initial prototype draft to address these needs from a technical perspective; (3) conducting think-aloud usability evaluations with representative rehabilitation professionals to identify additional requirements and refine the design; (4) testing the prototype in clinical simulation to assess workflow integration and safety; and (5) facilitating a final co-design workshop to converge feedback and generate a release-ready prototype. Because this study focused on a clinician-centered VGA system, the initial design task was to define the clinical outputs, interpretive support, and workflow requirements needed for routine CP gait assessment rather than to select between alternative prototype concepts [29]. Therefore, in this study, baseline clinical and workflow needs were first identified and used to develop a single needs-led prototype (design 1), which was then iteratively refined through subsequent co-design activities. In the text below, we explain how each step of the framework guided the co-design of the prototype gait analysis system to help CP rehabilitation (Figure 1).

### Step 1: Establish a Multidisciplinary Advisory Group

#### Composition and Roles of the Advisory Group

Developing a clinically useful gait analysis system for children with CP required input from clinical, technical, human factor, and visual design perspectives. We therefore purposively assembled a multidisciplinary advisory group to provide the expertise needed for formative co-design.

Clinical members were recruited from Shanghai Eber Medical Group, a specialist clinical network focused on neurological conditions and rehabilitation. The group operates rehabilitation hospitals in Shanghai, Qingdao, and Tianjin, and provides services for children with neurological conditions, including CP. This clinical network was selected because it represented a relevant early use setting for the prototype, with routine exposure to pediatric CP rehabilitation, gait assessment, treatment planning, and multidisciplinary clinical workflows. Clinical members were identified in consultation with clinical service leads. The purposive sampling criteria included professional role, clinical seniority, direct experience in CP gait assessment, involvement in rehabilitation planning, and familiarity with clinical workflow management. Based on these criteria, senior rehabilitation physicians and physical therapists from Shanghai Eber Medical Group were invited to join the advisory group.

The final advisory group comprised 17 members, including 12 senior rehabilitation professionals from Shanghai Eber Medical Group and 5 research and design members from the University of Auckland. The clinical members included 6 rehabilitation physicians and 6 therapists specialized in CP treatment. Most were service leads, therapy team leaders, senior therapists, or senior physicians. The University of Auckland members included 3 biomechanics researchers, 1 human factors engineer, and 1 graphic designer, who contributed expertise in gait analysis, prototype development, usability, workflow integration, and visual communication. Roles and responsibilities across different stages of work were explicitly allocated to support efficient and effective decision-making, as further detailed in Figure 1. Specifically, rehabilitation physicians and therapists served as representative end users and were engaged throughout the co-design process. In most steps, they acted as design partners, directly contributing to design decisions and shaping the system, except for step 2, where they participated as informants by providing ideas and feedback on the draft prototypes. The biomechanics researchers were responsible for the functional implementation of the prototype, including the specification and refinement of core functionalities across successive iterations while ensuring methodological rigor and translating clinical requirements into technically feasible solutions. In parallel, the human factors engineer focused on optimizing usability and workflow integration by revising the prototype through context-of-use analyses, heuristic evaluations, and iterative refinements. Complementing these efforts, the graphic designer enhanced the clarity and accessibility of the system by refining visual communication, interface layout, and report presentation.

#### Identifying Baseline Clinical Needs and System Requirements

In the predesign process, the baseline clinical needs and corresponding system requirements for a VGA system for children with CP were identified to establish an initial foundation for prototype development. These baseline requirements were not exhaustive or fixed, but served as a starting point that was progressively refined and expanded through subsequent co-design and usability activities.

Advisory group biomechanics researchers and rehabilitation professionals were involved in this stage. First, the 3 biomechanics researchers extracted and compared information from exemplar gait-analysis systems and reporting practices to provide design input for early prototype development. Specifically, information sources included published studies describing video-based or markerless gait-analysis systems, examples of conventional 3D gait analysis reports, and systems previously used or examined by the research team. For each source, the researchers recorded design-relevant information where applicable, including data input requirements, output parameters, report formats, interpretability, workflow demands, hardware or software requirements, and potential implementation barriers in routine clinical settings. The researchers then compared and consolidated the extracted information, grouped recurring design considerations into candidate requirement areas, and resolved differences through discussion. The extracted information was then synthesized into a preliminary list of candidate system requirements. Second, these candidate requirements were discussed in group meetings with clinical members from the advisory group, who contributed practical perspectives from routine CP rehabilitation workflows and highlighted additional considerations related to clinical interpretation, reporting needs, and workflow integration. The resulting requirements established a provisional baseline for the first prototype. Broader and more detailed end-user requirements were subsequently elicited and iteratively incorporated during later usability evaluations and co-design stages (steps 2 and 3), as additional stakeholders were engaged and system use scenarios became more clearly defined.

### Step 2: Develop Initial Drafts of the Prototype

Based on the baseline requirements identified in step 1, the first prototype (design 1) was developed by biomechanics researchers using BlazePose. BlazePose adopts a lightweight pose estimation algorithm (PEA), allowing convenient deployment on mobile or CPU-grade devices [30]. An automated rule-based framework was established to process PEA-derived key points from recorded videos, enabling functions like extracting gait cycles, calculating joint angles and spatiotemporal parameters, detecting gait deviations, and generating PDF reports. At this stage, emphasis was placed on implementing core functions, while aspects of usability and clinical integration were deferred to later iterations.

In the next stage, a human factors engineer reviewed design 1 through a usability heuristics evaluation (ie, 10 heuristics by Nielsen) [31] and a context-of-use analysis. This analysis examined factors affecting system integration, including intended users, tasks, physical environment, organizational setting (such as required clinical workflows and staffing roles), and technical constraints. This early expert-based review aimed to identify potential usability issues before involving representative end users. The identified issues and contextual considerations (not fully captured during step 1) were then incorporated into the system requirements to strengthen reliability and clinician confidence. The combined findings informed a refinement of design 2, which, with support from a graphic designer, incorporated usability principles and contextual considerations while retaining the core functional components of design 1.

### Step 3: Conduct Think-Aloud Usability Evaluations

After developing the prototype, we conducted online think-aloud formative usability sessions with 19 rehabilitation professionals from Shanghai Eber Medical Group in December 2024. These sessions were aimed at examining clinicians’ cognitive processes during interactions with the prototype and identifying usability barriers and their underlying causes among a small group of representative end users.

Participants were recruited from the group’s hospital sites in Shanghai, Qingdao, and Tianjin, using the purposive sampling criteria described in the *Composition and Roles of the Advisory Group* section. This sample included all 12 clinical members of the multidisciplinary advisory group and 7 additional rehabilitation professionals from the same clinical network. The additional 7 participants were included to broaden formative feedback beyond the core advisory group and to capture views from clinicians working across different hospital sites within the same rehabilitation network.

Each online session lasted approximately 60 minutes and was facilitated by the lead author (XG), a rehabilitation engineering researcher involved in prototype development. To reduce facilitator influence, all sessions followed the same task script, semistructured interview guide, and neutral prompting strategy. At the beginning of each session, the facilitator introduced the purpose of the evaluation, explained the think-aloud procedure, and reminded participants that the aim was to evaluate the prototype rather than their clinical performance. Participants were then asked to complete a set of typical clinical tasks using design 2. These tasks included reviewing a consistent CP gait case, opening or uploading the gait video in the prototype, running the video-based analysis, checking the pose-estimation and gait-cycle outputs, reviewing spatiotemporal parameters and joint-angle plots, interpreting automatically identified gait deviations, and reviewing the automatically generated report. During task completion, participants were instructed to verbalize their thoughts continuously while performing these tasks, following the concurrent think-aloud protocol. When participants became silent for more than a few seconds, the facilitator prompted them with neutral cues (eg, “please keep talking”) without directing their responses. All sessions were audio recorded, and the prototype interface was screen recorded to capture interactions. A second researcher (WL) attended the sessions as a nonintervening observer and took field notes on navigation difficulties, hesitation, points of confusion, interpretation difficulties, and workflow-related comments. Immediately after the tasks, participants filled out the System Usability Scale (SUS) questionnaire to rate the usability of the current prototype [32]. Subsequently, the moderator used a semistructured interview guide (see Multimedia Appendix 1) to clarify observations from the think-aloud exercise and to elicit further feedback on system functions, report interpretability, perceived benefits, and workflow compatibility.

The SUS questionnaire was scored using the standard 0‐100 scoring procedure. Overall usability was summarized as mean (SD), and individual item scores were inspected to identify aspects of the prototype that might require further refinement.

Feedback from the think-aloud exercise, semistructured interviews, and field notes was analyzed using a qualitative descriptive approach [33-35]. This approach was selected to provide a direct, low-inference summary of end-user feedback for formative prototype refinement. The lead author reviewed the recordings and field notes and organized participant feedback according to the main domains of the semistructured interview guide. Relevant feedback segments were descriptively labeled and grouped within these domains. Repeated comments were then summarized into perceived benefits and practical refinement categories, including functional expansion, report interpretability, and workflow compatibility. Additional end-user requirements were also identified to extend the baseline specifications defined in step 1. These findings informed subsequent prototype iterations, leading to design 3.

### Step 4: Test the Prototype in Clinical Simulation

To complement the usability evaluation in step 3, prototype design 3 was tested through in-person clinical simulations at Shanghai Eber Hospital, part of Shanghai Eber Medical Group, in August 2025 (see Figure 1). The aim of the clinical simulation was to assess workflow integration and explore use-related risks associated with prototype use in a realistic routine CP gait-assessment scenario. Rather than using separate risk-specific scenarios, the simulation explored potential risks as they arose during the routine video-based gait-assessment workflow.

Twelve rehabilitation professionals, including 6 physicians and 6 therapists, were recruited from Shanghai Eber Hospital, using the purposive sampling criteria described in the *Composition and Roles of the Advisory Group* section. Six of the 12 participants were members of the multidisciplinary advisory group and had also participated in the step 3 think-aloud usability evaluation. The remaining 6 were additional clinicians from Shanghai Eber Hospital, 2 of whom had also participated in step 3. Participants represented a range of professional seniority, with clinical experience spanning approximately 3 to 15 years in pediatric rehabilitation.

Simulations took place in their own clinical settings where care is routinely performed in order to maximize ecological validity. All child patients involved in the simulation were routine cases under the care of these clinicians. Parental informed consent was obtained for the use of their video data as part of the clinical simulation and research evaluation. Each clinician completed the same standardized scenario: assessing the gait of a child with CP as part of their routine rehabilitation session. The scenario was performed under 2 conditions: conventional observational gait analysis (OGA) and VGA using the prototype (design 3). To minimize sequence effects, the order of conditions was counterbalanced, with half of the participants (n=6) performing OGA first followed by the prototype, and the other half (n=6) completing the scenario in the reverse order. The inclusion of the conventional OGA condition was not intended to enable a quantitative comparison of diagnostic performance between OGA and VGA. Instead, it served as a familiar reference workflow to contextualize clinicians’ experiences with the prototype and to support the identification of workflow integration issues, interpretability challenges, and potential use-related risks that might not be evident in single-condition testing. In both conditions, participants carried out the full workflow, including video capture, gait analysis, and clinical interpretation of the results. All sessions were audio recorded, and structured field notes were taken to capture observations on workflow integration, system operation, clinical interpretation, and any patient physical safety–relevant issues. Following each pair of simulations, participants took part in a short debriefing discussion to reflect on their experience and highlight potential barriers or risks (see Multimedia Appendix 2 for topic guide questions in this discussion).

Data from the clinical simulations were analyzed using the same qualitative descriptive approach. The analysis was organized around the simulation objectives, including workflow integration, system stability, use-related physical safety, clinical interpretation risks, and prototype refinement needs. Field notes, recordings, and debriefing comments were reviewed by task stage, including video capture, system processing, report review, and clinical interpretation. Relevant observations and comments were extracted and mapped to the corresponding objective domains. Observed delays, system interruptions, error messages, technical-support needs, use errors, manual corrections, safety considerations during video capture, and interpretation-related concerns were summarized descriptively. The findings were then narratively summarized and used to guide subsequent prototype refinement, leading to design 4.

### Step 5: Generate a Final Prototype Using Co-Design Workshops

A co-design workshop was conducted with the advisory group and key stakeholders (ie, service leads and IT) to consolidate step 4 findings and prepare a release-candidate system. The workshop targeted consensus on three outputs: (1) the final prototype specification (report layout, parameter set and defaults, interaction wording, frozen algorithm/rule set, etc), (2) a standardized clinical workflow (roles, inputs/outputs, timing, and exception handling), and (3) an implementation guideline covering data capture and storage procedures, system operation guidelines, integration requirements, and training materials.

Sessions followed a progressive, item-by-item confirmation process. Each item represented a key component of the system specification, workflow, or implementation plan that had been refined during step 4. These items covered elements, such as report layout and terminology, parameter defaults, workflow roles and timing, data storage procedures, and user training materials. For each item, the facilitator presented the relevant evidence and proposed solution. Members then discussed and annotated the proposal within their respective disciplinary subgroups before reconvening in a plenary session to address any remaining disagreements or points requiring clarification. Agreement was recorded in a decision log, and unresolved items were iteratively refined until reaching ≥80% assent or resolved by chair adjudication in case of ties. This structured process ensured balanced input across clinicians, engineers, and service leads.

## Results

### Step 1 Outcomes: Baseline Clinical Needs and System Requirements Identified by the Advisory Group

The advisory group discussions, supported by a review of existing systems, identified a baseline set of clinical needs and corresponding system requirements for a VGA system in children with CP (Table 1). These centered on three domains: (1) system content, (2) system operation and workflow integration, and (3) clinical usability and support. Collectively, these baseline needs provided the foundation for developing the first prototype (design 1).

**Table 1.** Clinical needs, system requirements, and rationales identified across co-design steps 1‐3 for the prototype gait analysis system.

Clinical need	System requirement	Rationale
System content		
Objective gait quantification	Segmentation of gait cycles^a^ Automated extraction of spatiotemporal parameters (eg, step length and cadence)^a^ Addition of reference ranges to aid interpretation^b^	Provides objective data to complement observational gait assessment and contextualize severity.
Understanding kinematic contributors	Joint angle estimation across the gait cycle^a^ Generation of joint angle trajectories^a^ Addition of reference ranges to aid interpretation^b^	Enables clinicians to identify biomechanical sources of gait abnormalities.
Detection of gait abnormalities	Rule-based detection of clinically recognized deviations (eg, knee hyperextension and toe-walking)^a^ More granular categorization and severity indicators^b^	Supports targeted treatment planning and prioritization of interventions.
Standardized and interpretable reporting	Automated report summarizing results^a^	Facilitates integration of results into clinical documentation.
Comprehensive multiplane gait assessment	Add front-view video input and frontal-plane feature analysis^b^	Captures cerebral palsy gait deviations not visible from side view.
Alignment with established assessments	Automated conventional observational gait analysis scoring methods (eg, Edinburgh Visual Gait Score)^b^	Increases clinical acceptance by linking to existing scoring frameworks.
System operation and workflow integration		
Low-barrier data collection	Video capture using common devices^a^	Makes data acquisition feasible in routine outpatient settings.
Efficiency in processing	End-to-end video-to-report pipeline^a^	Reduces workload and supports timely clinical decision-making.
Feasible deployment in clinics	Operable on routine hardware (eg, smartphone, laptop, and tablet)^a^	Ensures smooth implementation in real-world practice.
Timely availability of results	Report generated within minutes^a^	Avoids workflow delays in busy clinical schedules.
Workflow compatibility	Reports formatted for easy incorporation into existing documentation systems (eg, electronic medical records or printed reports)^c^	Facilitates integration into clinicians’ existing routines without adding documentation burden.
Environmental robustness	Reliable performance under varied lighting and spatial conditions^c^	Ensures consistent accuracy and usability across real-world clinical environments.
Clinical usability and support		
Clinician-centered interpretability	Outputs designed with clear, standardized clinical terminology^a^ Consistency across reports to support communication^c^	Facilitates reliable interpretation and enables clinicians to explain results to families within routine practice.
Ease of use and navigation	Intuitive interface, streamlined navigation^c^	Improves efficiency and reduces cognitive load for clinicians.
Clear visualization of results	Basic charts included^a^ Request for clearer graphics, color coding^c^	Makes outputs easier to interpret immediately.
Error prevention and feedback	Requests for quality warnings^c^	Reduces the risk of misinterpretation due to poor data quality.
System feedback and responsiveness	Visible confirmation of each operation (eg, completion messages and progress indicators)^c^	Maintains confidence that each operation has been executed correctly.
Training and support materials	Provision of training materials and workflow guidelines^b^	Standardizes use and supports clinician adoption.

a Baseline requirement (step 1).

b Additional requirement identified during usability evaluations (step 3).

c Added after heuristic/context-of-use analysis (step 2).

### Step 2 Outcomes: Initial Drafts of the Prototype

The baseline clinical needs and system requirements identified in step 1 guided the development of the first draft prototype (design 1). Emphasis at this stage was placed on implementing system core functions, including visualization of pose recognition, automated and manual gait cycle segmentation, calculation of spatiotemporal parameters, estimation of joint angles, rule-based detection of selected gait deviations, and automated generation of a summary PDF report. The system architecture and algorithmic choices were made based on clinical feasibility and interpretability. MediaPipe BlazePose was selected for pose estimation due to its lightweight implementation, sufficient accuracy for sagittal-plane analysis on standard CPUs [36], and ability to provide foot key-point estimates that enable the assessment of foot and ankle abnormalities. Spatiotemporal and joint angle calculations followed standard biomechanical definitions to ensure clinical relevance, while deviation detection rules were derived from published CP gait literature and refined with input from experienced clinicians.

A subsequent heuristic evaluation and context-of-use analysis of design 1 identified additional usability and contextual requirements beyond those captured in step 1 (Table 1). The heuristic review (based on the 10 principles by Nielsen [31]) revealed several issues related to system safety and transparency, such as the need for clear feedback after each operation and warnings when errors occurred. In parallel, the context-of-use analysis (key components summarized in Multimedia Appendix 3) highlighted practical considerations for real-world clinical deployment, including the range of intended users, routine clinical environments, typical patient presentations, and technical constraints. These findings informed the refinement of design 1 (design 2; see Multimedia Appendix 4), which incorporated visual hierarchy through standardized font size, color, and layout adjustments, along with real-time visual feedback and warning prompts for error prevention. To enhance scalability, the system was also deployed as a web-based interface accessible through a simple URL, enabling easy use without local installation. With these refinements, design 2 emerged as an initial draft prototype that was technically stable and ready for subsequent end-user evaluations. Examples of these refinements are provided in Multimedia Appendix 3.

### Step 3 Outcomes: Usability Findings and Prototype Refinements

#### Overall Usability Findings

Nineteen rehabilitation professionals participated in the think-aloud usability session, followed by the SUS questionnaire and interviews. The prototype (design 2) achieved a mean SUS score of 75.8 (out of 100; SD 7.5), indicating good perceived usability according to established SUS interpretive benchmarks [37]. A qualitative descriptive approach identified 2 broad categories: perceived benefits and improvement needs (see Table 1). Improvement needs were further organized into functional, interpretability, and workflow improvement.

#### Perceived Benefits

Overall, participants reported that the system could be useful and expressed interest in applying it in treatment preparation. They felt that the system could (1) support OGA by allowing slow-motion review of joint movements and gait cycles, (2) provide meaningful deviation identification through automated detection and visual display of abnormal patterns, and (3) assist in evaluating treatment outcomes by enabling comparison of pre- and postintervention gait performance. Some participants also praised the ease of use of the application, noting that the interface was intuitive and straightforward even for users who were not proficient with digital or smartphone-based systems.

#### Improvement Needs

For functional expansion, participants noted that the system recognizes only a limited range of gait deviations and suggested expanding the deviation-detection library to cover a broader set of CP-specific gait patterns. They also recommended incorporating coronal plane analytical functions, as movements, such as hip abduction, adduction, and pelvic rotation, are clinically important for evaluating rehabilitation outcomes in children with CP. In terms of interpretability, participants reported that while the system could identify deviations, additional clinical explanations and normative references were needed to clarify what each deviation meant for the patient’s function. They suggested integrating explanatory notes and annotated normal ranges within the report to assist clinical interpretation. Regarding the workflow, therapists pointed out that the system’s accuracy could be affected by participant clothing and suboptimal subject positioning within the camera frame. They proposed developing standardized guidelines and simplifying data collection procedures to ensure consistency across different clinical settings. Corresponding modifications were implemented in design 3, as summarized in Table 2, to enhance the system’s analytical scope, interpretability, and practical integration into rehabilitation workflows.

**Table 2.** Usability analysis for design 2 and resulting system modifications in step 3 (n=19).

Theme, category, and topic	Illustrative quotes^a^	Summary of feedback	System modification
Perceived benefits			
Usefulness		Participants generally described the system as useful.	
Support observational gait analysis	“What I use most is generating the skeletal model and then using the slow-motion or pause functions to observe specific moments. For example, during stance, to see whether the knee is fully extended or how much it bends, and whether the foot angle is correct at the instant of leg extension.”		—^b^
Provide useful deviation identification	“It’s pretty helpful that the system can identify gait deviations. It helps us sort patients into different categories and look into specific problems more closely.” “Overall, it does a good job. The deviations it shows are mostly right and match what I usually see, so it’s really helpful in understanding the patient’s gait.”		—
Evaluate treatment outcomes	“It’s also helpful for checking treatment results, like after surgery or when kids are discharged. By comparing the first and last assessments, we can easily see how much they’ve improved.”		—
Ease of use		Participants generally described the system as easy to use.	
Easy to use the system	“It’s pretty easy to use, even for someone like me who’s not very tech-savvy.” “I think the system is quite straightforward. I didn’t need much time to figure it out.” “The system interface is easy to use and very clear. There are also hints that guide me on what to do next.”		—
Improvements/limitations			
Functional improvement		—	
More gait deviations	“Right now, it only detects a few common gait issues. If it could identify more types of deviations specific to CP^c^, that’d be even better.”		Expand the deviation library for more CP-specific gait patterns.
Add coronal parameters	“Things like pelvic rotation or hip abduction and adduction are really important for CP rehab. It’d be nice if the system could also look at movements from the coronal view, not just the side.”		Integrate the coronal plane input and analytical functions.
Interpretability improvement		—	
Access to cause-related knowledge	“Sometimes it shows a deviation, but we’d like more clinical explanation, like what that actually means for the patient’s function.”		Provide explanatory information linking identified deviations to potential underlying causes such as muscle weakness or spasticity.
Add comparison features	“If it could show what the normal range looks like, it’d be easier for us to tell how much the patient differs from it.”		Include annotations of normal reference ranges to assist clinicians in evaluation.
Add summary scoring for clinical reference	“We often use observation-based scales in clinic, so if the system could provide something like that automatically, it’d be really helpful.”		Implement automated clinical scales (eg, Edinburgh Visual Gait Score) to quantify gait deviation severity.
Workflow improvement		—	
Video capture guideline	“Sometimes the system can’t recognize well if participants wearing loose or if the participant’s full movement is not properly framed. It’d be great to have a clear guide for data collection.”		Develop standardized and streamlined data collection protocols to ensure accuracy while maintaining ease of implementation in clinical practice.
Simplified data collection	“In the clinic, we don’t always have perfect space and lighting and enough time. It’d help if the system could work with simpler setups.”		Develop standardized and streamlined data collection protocols to ensure accuracy while maintaining ease of implementation in clinical practice.

a The illustrative quotes represent participants’ typical responses; original interviews were conducted in Chinese and translated into English for reporting.

b Not applicable.

c CP: cerebral palsy.

Beyond usability aspects, the interviews revealed participants’ broader expectations as end users, offering insights into preferred report content, workflow alignment, and information prioritization. These expectations were incorporated into the system requirements, as summarized in Table 1.

### Step 4 Outcomes: Clinical Simulation Findings and Prototype Optimization

Clinical simulations were conducted in real clinical settings to evaluate workflow integration, safety, and potential use-related risks associated with the prototype (design 3). The main findings are summarized in Table 3.

**Table 3.** Examples and key findings of the analysis of the simulations.

Example of data analysis	Key findings
Workflow integration: Use of conventional observational gait analysis as a familiar reference workflow to contextualize prototype use and identify whether physicians/therapists can naturally use the system within their regular work rhythm	Most participants were able to complete the full workflow using the prototype without additional assistance. Participants perceived that, relative to their usual observational gait analysis workflow, the prototype could reduce manual notetaking and interpretation time while providing additional quantitative parameters to support clinical interpretation.
System stability and quality-control issues: Observation of system failures, crashes, processing interruptions, delays, error messages, and manual corrections during gait-event detection and gait-deviation identification.	No system failures, crashes, or processing interruptions were observed. There were 13 functional errors in recorded observations (7 in gait event identification from the side video and 6 in gait deviation identification from the front video). There were 4 corrections defined as cases where the original incorrect mark/value was changed to the correct value.
Use-related physical safety: Observation of any safety-related events or patient instability during gait data capture, and analysis of clinicians’ feedback regarding supervision requirements and safe recording setup.	All simulations were conducted safely under therapist supervision. However, several clinicians stressed the importance of having clear safety guidelines during data capture, particularly for children with balance difficulties, to reduce the risk of falls. In some sessions, minor adjustments to the environment or therapists’ position were needed to maintain the child’s stability.
Clinical interpretation risks: Qualitative analysis of recordings, field notes, and debriefings to identify interpretation-related risks during clinical use.	Participants highlighted potential risks of overreliance on automatically generated outputs and inconsistent interpretation of deviation labels due to differences in clinical background and judgment.
Suggested areas of improvement: Qualitative analysis of recordings, field notes, and debriefings to synthesize participants’ suggestions for prototype improvement.	Suggested refinements included combining automatic and manual segmentation, revising the report layout for clarity, and providing a concise user guide for standardized operation.

One key discussion point was the system’s practicality in real clinical workflows. Clinicians confirmed that the prototype could be operated independently without technical support, which streamlined the gait assessment process by reducing manual notetaking and interpretation time. The system functioned stably throughout all simulations, and no safety incidents occurred. Nonetheless, given the inherent fall risk for patients with balance difficulties during gait assessment, clinicians emphasized the need for adaptive safety guidelines and flexible supervision protocols during video capture. This flexibility is necessary to allow for immediate adjustments, such as micro-adjustments to the environment or the therapist’s position, ensuring patient safety across varied clinical settings. Another major point concerned the interpretability of automatically generated reports. Participants noted occasional differences in how deviation labels were understood and highlighted the risk of overreliance on automated outputs.

The simulation findings collectively informed the optimization of the prototype, leading to design 4, which incorporated combined automatic-manual segmentation, a clearer and more intuitive report layout, and a concise user guide with safety instructions to support standardized operation (see Multimedia Appendix 4).

### Step 5 Outcomes: Final Prototype Generation

The co-design workshop consolidated the findings from the clinical simulation and resulted in consensus across the multidisciplinary advisory group on the final system specification, standardized workflow, and implementation guideline.

**Figure 2.**
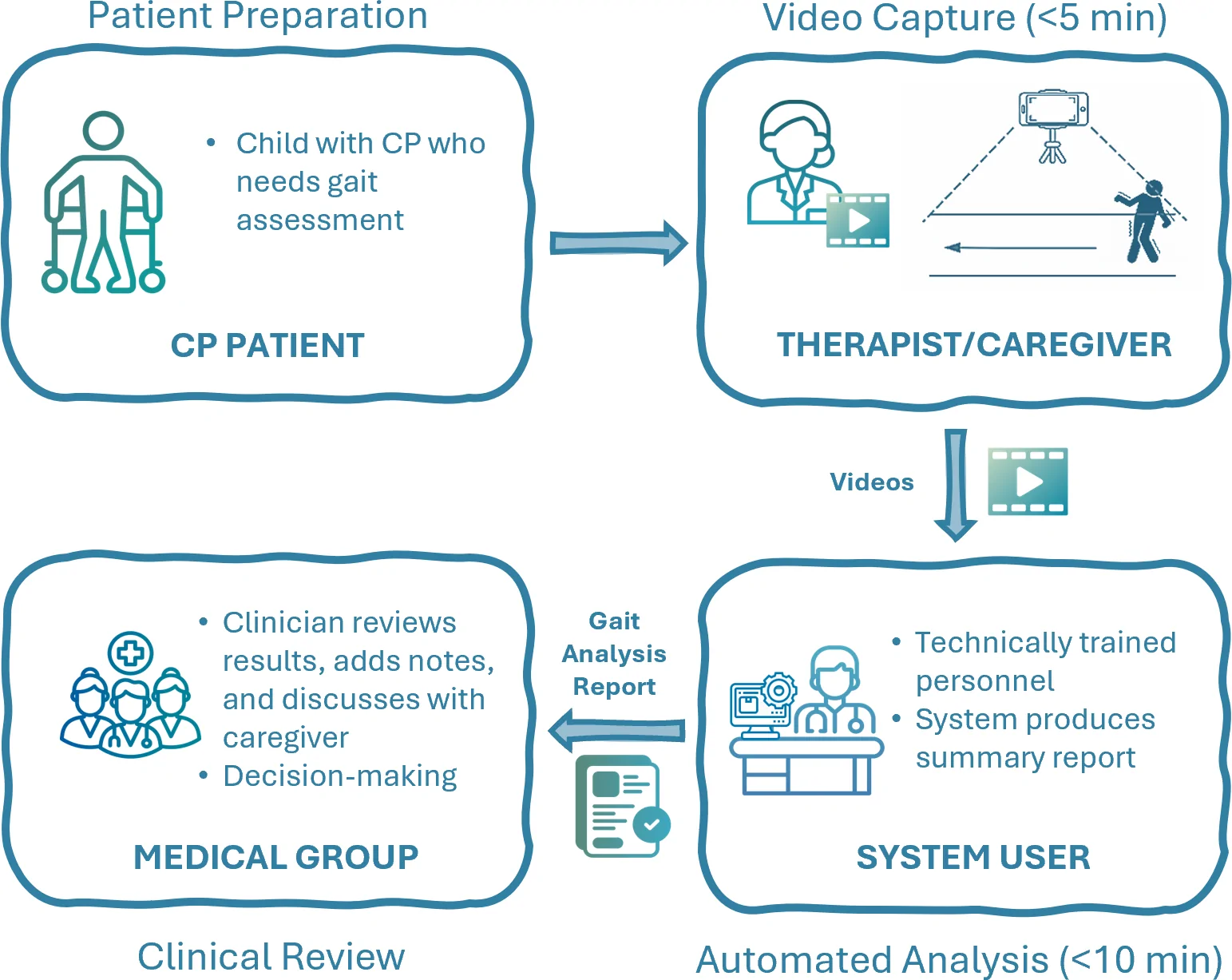
Clinical workflow integration of the video-based gait analysis system. CP: cerebral palsy.

The group confirmed the final prototype (design 5; see Multimedia Appendix 5), which retained all validated analytical functions while incorporating refinements to report layout, terminology, and visual hierarchy for improved clarity and interpretability. Agreement was also reached on a standardized clinical workflow detailing roles, inputs, and timing to ensure consistent use across settings. Figure 2 illustrates how clinicians use the VGA system during routine consultations, from video capture and automated analysis to report generation and interpretation. In parallel, a comprehensive implementation guideline was developed, covering data capture and storage procedures, integration with existing documentation systems, and concise training materials for clinical staff (see Multimedia Appendix 6). The workshop achieved high agreement among participants (≥80%) on all key items, completing the co-design phase and preparing the system for large-scale evaluation.

As the system is designed for Chinese-speaking clinicians, the operational interface of the deployed system is presented in Chinese. For this manuscript, the interface screenshots in Multimedia Appendix 5 have been translated into English and are provided for illustrative display only.

## Discussion

### Principal Findings

This study applied a structured co-design framework (FRESCO) to develop and iteratively refine a VGA system tailored for the clinical assessment of children with CP. Beyond the development of the prototype itself, the study also examined how a structured co-design process can support the translation of a research-based VGA system into a clinician-centered decision-support tool. In doing so, it makes three design-oriented contributions: (1) establishing baseline clinical and workflow requirements before prototype refinement; (2) translating VGA-derived kinematic parameters into clinician-centered and decision-relevant outputs; and (3) incorporating multidisciplinary feedback and clinical workflow simulation into iterative refinement of usability, safety, and implementation fit. Through 5 progressive steps, the system evolved from a conceptual prototype into a release-candidate decision-support tool (final prototype) that is technically stable and ready for large-scale clinical evaluation. This formative process demonstrated how multidisciplinary collaboration among rehabilitation clinicians, engineers, and biomechanics experts can translate clinical requirements for gait assessment into an interpretable and workflow-compatible digital solution.

Several findings were directly supported by the current formative data. Across the context-of-use analysis, think-aloud usability evaluation, and clinical simulation, clinicians emphasized the importance of workflow fit, low operational burden, interpretable outputs, and retaining professional control over final clinical interpretation. Building on these findings, we derived two data-informed design principles that may be transferable to other clinician-centered digital health systems: (1) supporting workflow-compatible implementation by minimizing technical and operational barriers, and (2) prioritizing clinical autonomy and actionability. As these principles were developed from a single-site formative co-design study, they are intended to provide practical reference points for future system development rather than definitive implementation rules.

The first principle, supporting workflow-compatible implementation by minimizing technical and operational barriers, was grounded in findings from the context-of-use analysis and clinical simulation. These findings showed that clinicians needed a system that could fit within routine outpatient workflows without requiring dedicated hardware, complex setup procedures, software installation, or specialized training in motion capture. Unlike traditional marker-based motion capture systems that require specialized laboratory environments, controlled setups, and lengthy postprocessing [6], our system was intentionally designed for use in routine outpatient settings. By relying on routine video recordings without introducing new data acquisition procedures, the system integrates directly into established assessment workflows. To further minimize technical and logistical barriers to adoption, the system can be deployed in a lightweight, web-based format, allowing flexible access via a simple URL [38,39]. This design choice allowed the system to function within the existing physical and technological infrastructure of typical rehabilitation clinics. Moreover, the end-to-end video-to-report pipeline ensures that clinicians can capture, process, and interpret gait data within the time constraints of a standard consultation, thereby supporting seamless integration into clinical workflows.

The second principle, prioritizing clinical autonomy and actionability, was grounded in findings from the think-aloud usability evaluation and clinical simulation. Participants valued clinically meaningful and interpretable outputs, but the simulation also highlighted the need to avoid overreliance on automated outputs and to preserve clinicians’ control over the final interpretation. In contrast to systems that primarily present final predictions without explicit representation of intermediate analytical steps [17,40,41], the proposed system was intentionally designed to support stepwise clinical reasoning rather than automated decision-making. Specifically, system outputs are structured to align with clinicians’ established reasoning processes, progressing from raw kinematic information to identified gait deviations and, where appropriate, to potential contributing biomechanical or neuromuscular factors. This transparent, staged representation enables clinicians to evaluate, contextualize, and, if necessary, challenge system-generated assessments based on their professional expertise and patient-specific knowledge. By providing interpretable and clinically contextualized outputs within a stepwise decision-support pipeline, the system facilitates informed clinical judgment while preserving clinicians’ control over final interpretation and decision-making. In this way, clinical autonomy is supported through meaningful engagement with the reasoning process underlying system outputs. This design perspective aligns with the sociological and informatics accounts of clinical autonomy, which emphasize the need for clinicians to retain interpretive control over diagnostic and evaluative processes through access to transparent and interpretable reasoning pathways, rather than being limited to opaque, outcome-only system outputs that obscure how assessments are derived [42,43].

Furthermore, the study also highlighted how combining co-design with a mixed methods approach helped strengthen both the design process and the evaluation outcomes. Specifically, the integration of iterative qualitative insights with quantitative usability metrics (eg, SUS scores) provided systematic triangulation, enabling the active engagement of clinicians to mutually inform experiential feedback and quantitative results. This ensured design decisions remained closely aligned with complex clinical practice and contributed directly to the system’s high usability and workflow compatibility, leading to more robust and generalizable findings [44,45].

The formative usability evaluation (step 3) confirmed a good usability score (SUS score: mean 75.8, SD 7.5), while detailed item analysis revealed 2 items with notably lower ratings: “I need the support of a technical person to be able to use this system” and “I need to learn a lot of things before I could get going with this system.” These responses suggest that while the prototype was intuitive in operation, participating clinicians and therapists still lacked technical and analytical confidence in interpreting gait data. This observation aligns with previous reports that the main barrier to using quantitative gait analysis technologies in practice is clinicians’ limited data expertise and difficulty interpreting kinematic information [16,46-48]. Recent evidence suggests that providing clinicians with structured reports, visual summaries, and key result highlights can improve interpretability and facilitate clinical adoption of gait technologies [11]. Accordingly, in addition to presenting relevant quantitative parameters and plots, our system reports were designed to place particular emphasis on interpretable outputs. The reports present clinically meaningful information, including normative references, identified gait deviations with severity ratings, and potential contributing factors inferred from the knowledge base. In addition, an implementation guideline was developed to specify user roles in data capture, video processing, report generation, and clinical interpretation. Within this guideline, it is recommended that health care institutions assign technically trained personnel to manage video processing and report generation, as these steps require familiarity with the software workflow and quality-control procedures. This role allocation is intended to support consistent report generation and reduce the operational burden on front-line clinicians, while clinicians retain responsibility for interpreting the full report, including quantitative parameters, visual plots, identified gait deviations, and explanatory information. Structured predeployment training sessions are also included in the implementation guideline to enhance data literacy and ensure the system’s effective and reliable use within routine clinical workflows.

The clinical simulation (step 4) revealed a potential factor that could influence the system’s practical effectiveness: variability in how clinicians interpret automatically identified gait deviations. Although explanatory notes were provided in the prototype to link each deviation with its possible biomechanical or neuromuscular causes and thereby enhance interpretability, clinical interpretation ultimately depends on the clinician’s professional knowledge and holistic understanding of the patient’s condition. Identifying the potential contributors to gait deviations can only offer supplementary insights for clinicians in terms of subsequent treatment planning and the evaluation of therapeutic outcomes [49]. Previous OGA studies have reported variability in clinicians’ interpretation of the same gait data/outputs [50,51]. Although automated or semiautomated gait assessment systems are designed to standardize outputs, differences in how clinicians interpret these outputs remain a potential source of variability [52]. Therefore, even when the system provides indicators or explanatory cues, its reliability in supporting clinical decision-making may still be constrained if clinicians interpret those cues inconsistently. These findings highlight the importance of supporting clinicians’ understanding of system outputs and underlying reasoning processes to promote consistent interpretation and effective clinical use.

### Limitations and Directions for Future Research

This study has several limitations. First, the sample of participating clinicians was geographically limited to a single clinical group, which may restrict the generalizability of the findings. Second, the system’s reliance on a rule-based algorithm for deviation-cause mapping inherently limits its algorithmic precision and robustness, potentially resulting in a struggle to accurately interpret complex or atypical CP gait patterns. Third, the system’s explanatory and reporting functions were evaluated qualitatively rather than quantitatively. This study also did not formally compare different report formats or levels of explanatory detail, which may influence clinicians’ efficiency, trust, and interpretation consistency. Fourth, children with CP and their caregivers or family members were not included as co-design participants in this study. The main reason for this decision was that the prototype was clinician-centered and was designed primarily as a decision-support tool for rehabilitation professionals, rather than as a patient- or caregiver-facing system. Nevertheless, the perspectives of patients and caregivers should not be overlooked, as they may influence the acceptability, feasibility, and ethical implementation of VGA in routine care, particularly in relation to video data capture, privacy, and communication of assessment results.

Building on these findings, future work should focus on 3 main priority areas. First, multisite trials are essential to evaluate the system’s generalizability, interrater reliability, and impact on clinical decision-making and treatment outcomes. Second, efforts must be directed toward algorithmic and interpretive enhancement: expanding the knowledge base to integrate probabilistic reasoning or machine-learning models could enhance the precision of deviation-cause mapping. To mitigate the critical risk of variability in clinical interpretation, future evaluations should also compare different report formats and levels of explanatory detail to determine how best to balance efficiency, interpretability, clinical justification, and avoidance of overreliance on automated outputs. Finally, in terms of implementation and training frameworks, establishing shared interpretation frameworks is key to maximizing clinical benefit. This requires developing structured training modules focusing on standardized interpretation, embedding clinician feedback loops and uncertainty indicators within the report interface, and incorporating patient and caregiver perspectives to support broader implementation, particularly around acceptability, privacy, and communication of results.

### Conclusion

Using the FRESCO co-design framework and iterative collaboration among clinicians, engineers, and biomechanics experts, we progressively refined a clinically oriented VGA system for children with CP into a stable and interpretable decision-support tool that demonstrated good usability and workflow compatibility. The study highlights how clinician-centered, structured co-design can facilitate the creation of digital tools that are both technically feasible and aligned with clinical reasoning. Beyond the immediate context of CP, the transferable design principles identified in this work, emphasizing interpretability, workflow integration, and low-barrier data capture, may inform the development of other clinically relevant digital health systems.

## Supplementary material

10.2196/92823 Multimedia Appendix 1 System Usability Scale questionnaire and semistructured interview guide used to follow the think-aloud exercises.

10.2196/92823 Multimedia Appendix 2 Examples of topic guide questions used in the postsimulation focus group.

10.2196/92823 Multimedia Appendix 3 Context-of-use analysis and design refinements for the system.

10.2196/92823 Multimedia Appendix 4 Examples of the initial draft of the prototype (design 2).

10.2196/92823 Multimedia Appendix 5 Example of the final prototype (design 5). The panels (A-F) illustrate the core functional modules of the system: (A) Basic information and side video pose estimated; (B) Gait event identification and gait cycle extraction; (C) Spatiotemporal parameter and joint angle calculations; (D) Front video input and analysis; (E) Automated Edinburgh Visual Gait Score scoring; (F) Gait deviation identification and potential causes reasoning. The original system interface is in Chinese; indicative English translations have been provided here for the purpose of this publication.

10.2196/92823 Multimedia Appendix 6 Implementation guideline of the gait analysis system.

## Abbreviations

AHREC: Auckland Health Research Ethics Committee
CP: cerebral palsy
FRESCO: Framework for Co-design of Clinical Practice Tools
OGA: observational gait analysis
PEA: pose estimation algorithm
SUS: System Usability Scale
UCD: user-centered design
VGA: video-based gait analysis
