# Supplementary material for: Co-Designing a Clinician-Centered Video Gait Assessment System for Cerebral Palsy Based on the Framework for Co-Design of Clinical Practice Tools: Iterative Development and Formative Evaluation Study
Source: J Med Internet Res. 2026 Aug 21;28:e92823. doi: 10.2196/92823 (PMC13496319; doi:10.2196/92823)
Supplement: Supplementary file 1 [file jmir-v28-e92823-s005.pdf]

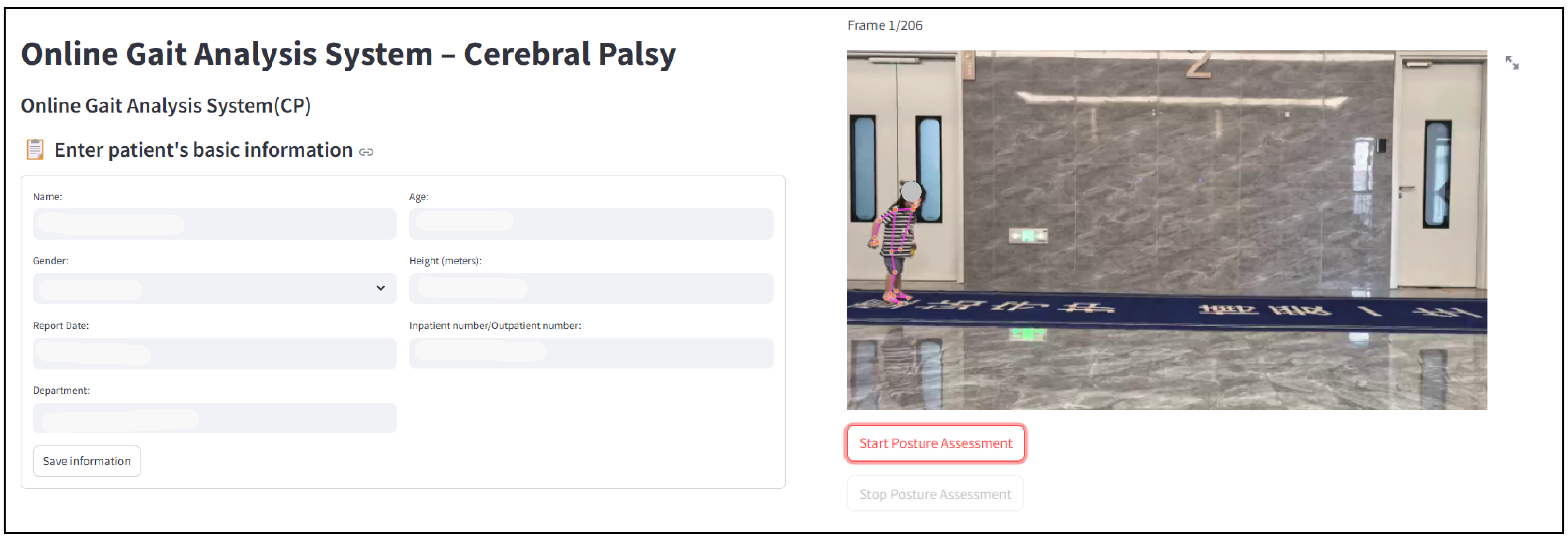
Online Gait Analysis System – Cerebral Palsy
Online Gait Analysis System(CP)
Enter patient's basic information
Name:
Age:
Gender:
Height (meters):
Report Date:
Inpatient number/Outpatient number:
Department:
Save information
Frame 1/206
Start Posture Assessment
Stop Posture Assessment

A.

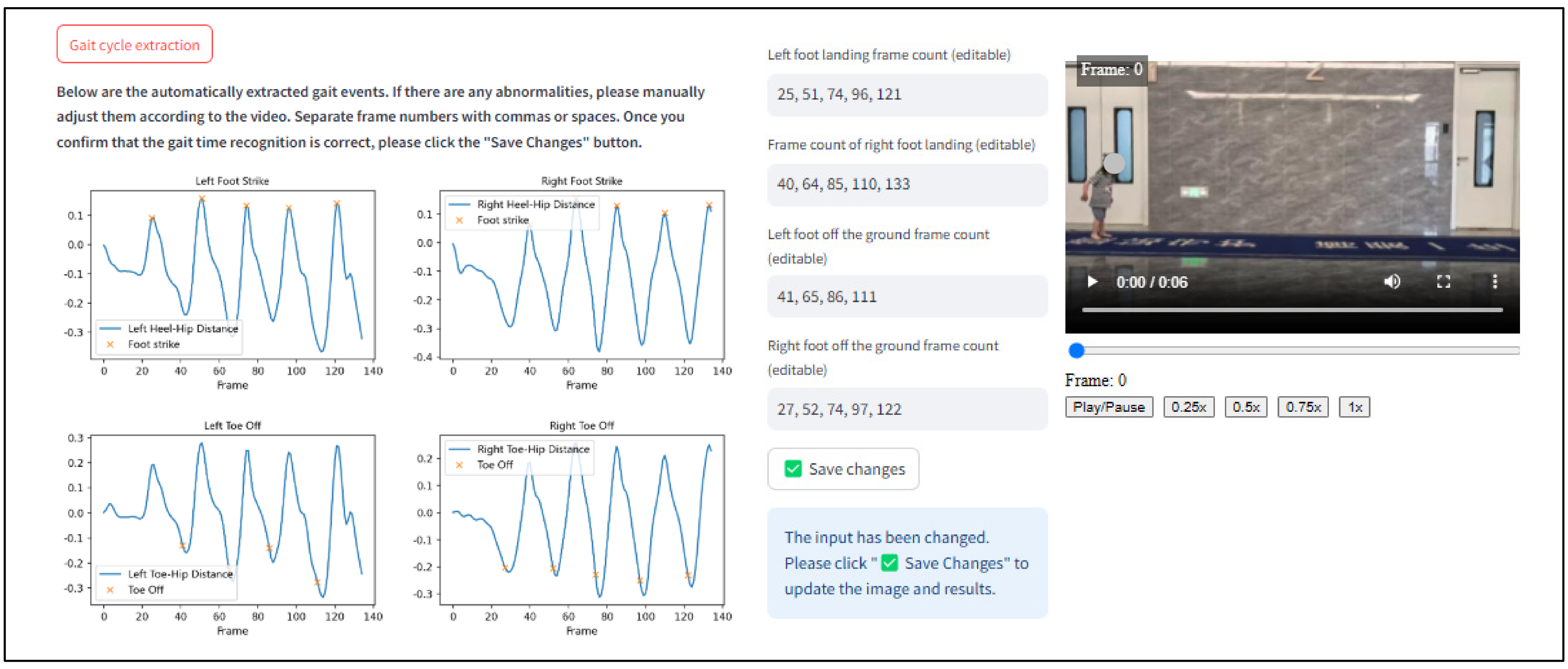
Gait cycle extraction
Below are the automatically extracted gait events. If there are any abnormalities, please manually adjust them according to the video. Separate frame numbers with commas or spaces. Once you confirm that the gait time recognition is correct, please click the "Save Changes" button.
Left Foot Strike
Left Heel-Hip Distance
Foot strike
Right Foot Strike
Right Heel-Hip Distance
Foot strike
Left Toe Off
Left Toe-Hip Distance
Toe Off
Right Toe Off
Right Toe-Hip Distance
Toe Off
Frame
Left foot landing frame count (editable)
25, 51, 74, 96, 121
Frame count of right foot landing (editable)
40, 64, 85, 110, 133
Left foot off the ground frame count (editable)
41, 65, 86, 111
Right foot off the ground frame count (editable)
27, 52, 74, 97, 122
Save changes
The input has been changed. Please click " Save Changes" to update the image and results.
Frame: 0
0:00 / 0:06
Frame: 0
Play/Pause
0.25x
0.5x
0.75x
1x

B.

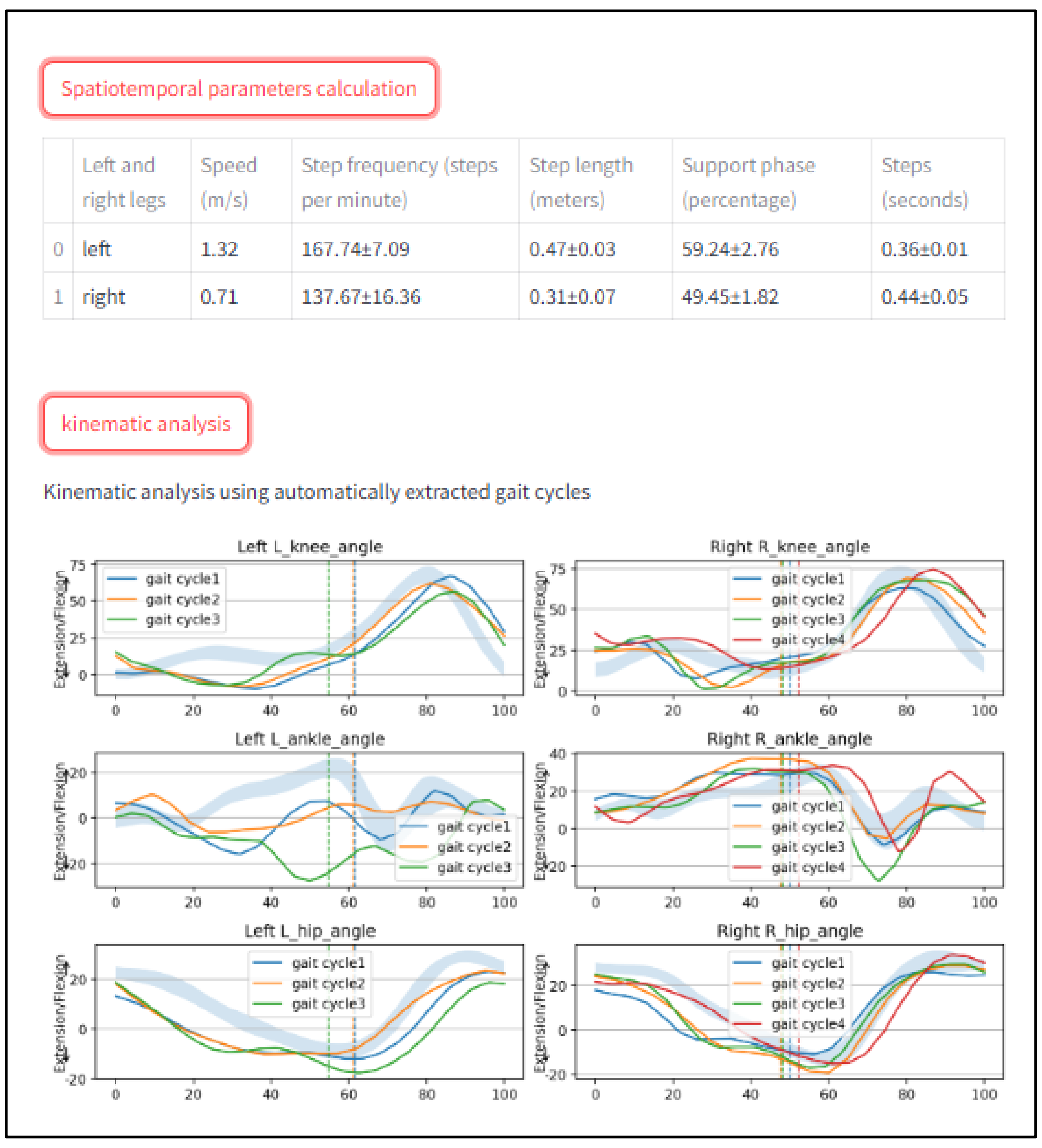
Spatiotemporal parameters calculation
Left and right legs | Speed (m/s) | Step frequency (steps per minute) | Step length (meters) | Support phase (percentage) | Steps (seconds)
0 left | 1.32 | 167.74±7.09 | 0.47±0.03 | 59.24±2.76 | 0.36±0.01
1 right | 0.71 | 137.67±16.36 | 0.31±0.07 | 49.45±1.82 | 0.44±0.05
kinematic analysis
Kinematic analysis using automatically extracted gait cycles
Left L_knee_angle
Right R_knee_angle
Left L_ankle_angle
Right R_ankle_angle
Left L_hip_angle
Right R_hip_angle
gait cycle1
gait cycle2
gait cycle3
gait cycle4
Extension/Flexion

C.

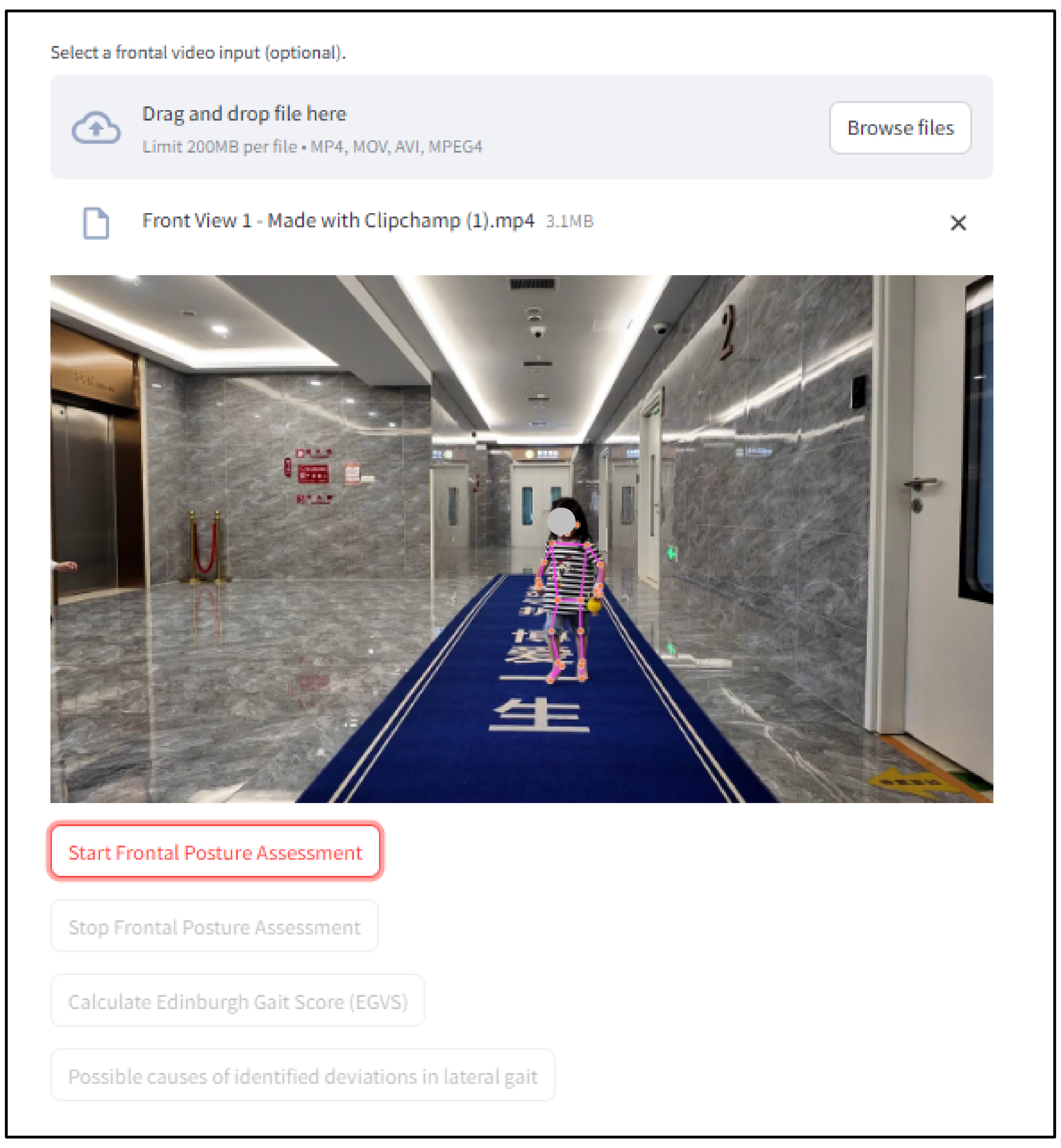
Select a frontal video input (optional).
Drag and drop file here
Limit 200MB per file • MP4, MOV, AVI, MPEG4
Browse files
Front View 1 - Made with Clipchamp (1).mp4 3.1MB
Start Frontal Posture Assessment
Stop Frontal Posture Assessment
Calculate Edinburgh Gait Score (EGVS)
Possible causes of identified deviations in lateral gait

D.

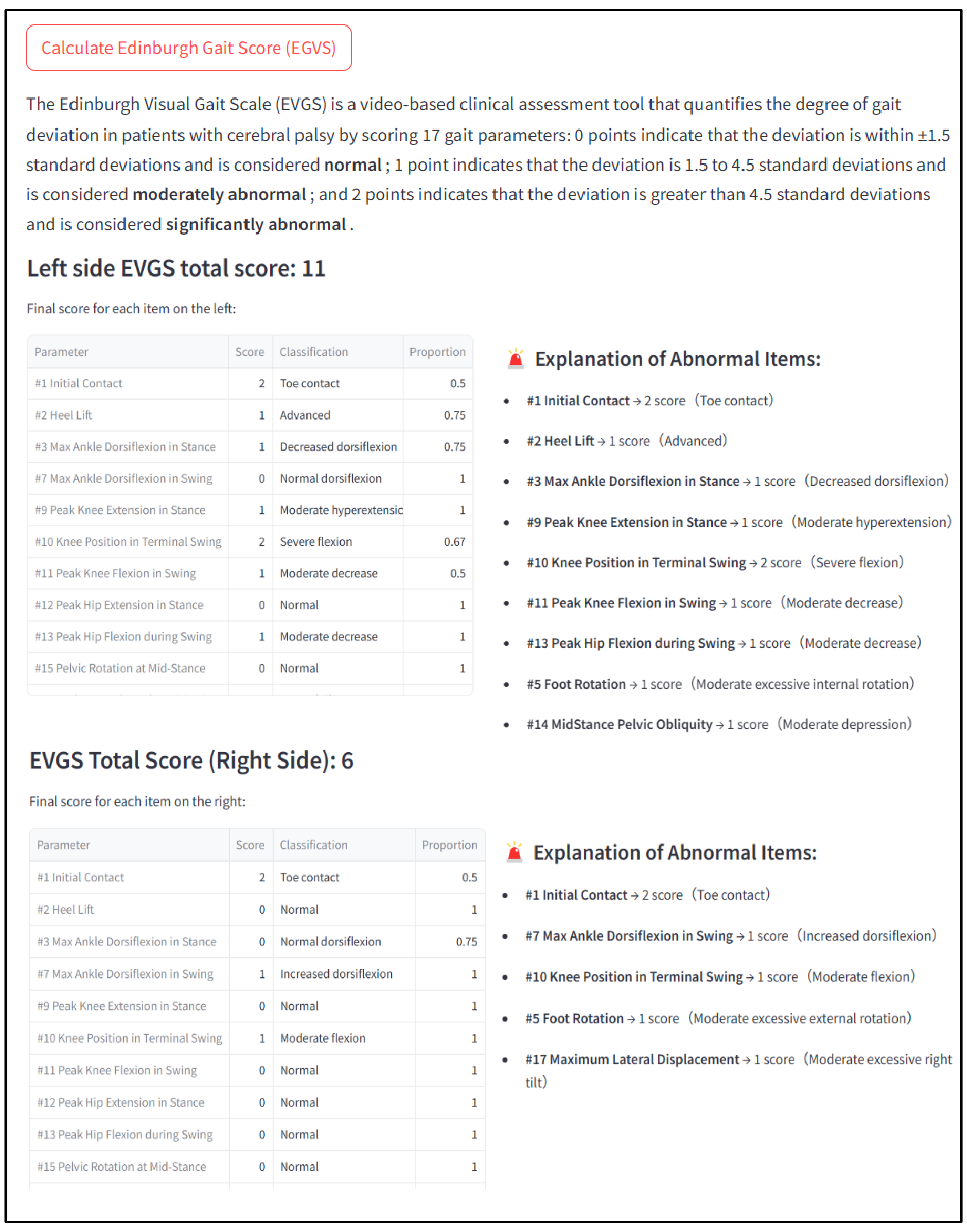
Calculate Edinburgh Gait Score (EGVS)
The Edinburgh Visual Gait Scale (EVGS) is a video-based clinical assessment tool that quantifies the degree of gait deviation in patients with cerebral palsy by scoring 17 gait parameters: 0 points indicate that the deviation is within ±1.5 standard deviations and is considered normal; 1 point indicates that the deviation is 1.5 to 4.5 standard deviations and is considered moderately abnormal; and 2 points indicates that the deviation is greater than 4.5 standard deviations and is considered significantly abnormal.
Left side EVGS total score: 11
Final score for each item on the left:
Parameter | Score | Classification | Proportion
#1 Initial Contact | 2 | Toe contact | 0.5
#2 Heel Lift | 1 | Advanced | 0.75
#3 Max Ankle Dorsiflexion in Stance | 1 | Decreased dorsiflexion | 0.75
#7 Max Ankle Dorsiflexion in Swing | 0 | Normal dorsiflexion | 1
#9 Peak Knee Extension in Stance | 1 | Moderate hyperextensic | 1
#10 Knee Position in Terminal Swing | 2 | Severe flexion | 0.67
#11 Peak Knee Flexion in Swing | 1 | Moderate decrease | 0.5
#12 Peak Hip Extension in Stance | 0 | Normal | 1
#13 Peak Hip Flexion during Swing | 1 | Moderate decrease | 1
#15 Pelvic Rotation at Mid-Stance | 0 | Normal | 1
Explanation of Abnormal Items:
#1 Initial Contact → 2 score (Toe contact)
#2 Heel Lift → 1 score (Advanced)
#3 Max Ankle Dorsiflexion in Stance → 1 score (Decreased dorsiflexion)
#9 Peak Knee Extension in Stance → 1 score (Moderate hyperextension)
#10 Knee Position in Terminal Swing → 2 score (Severe flexion)
#11 Peak Knee Flexion in Swing → 1 score (Moderate decrease)
#13 Peak Hip Flexion during Swing → 1 score (Moderate decrease)
#5 Foot Rotation → 1 score (Moderate excessive internal rotation)
#14 MidStance Pelvic Obliquity → 1 score (Moderate depression)
EVGS Total Score (Right Side): 6
Final score for each item on the right:
Parameter | Score | Classification | Proportion
#1 Initial Contact | 2 | Toe contact | 0.5
#2 Heel Lift | 0 | Normal | 1
#3 Max Ankle Dorsiflexion in Stance | 0 | Normal dorsiflexion | 0.75
#7 Max Ankle Dorsiflexion in Swing | 1 | Increased dorsiflexion | 1
#9 Peak Knee Extension in Stance | 0 | Normal | 1
#10 Knee Position in Terminal Swing | 1 | Moderate flexion | 1
#11 Peak Knee Flexion in Swing | 0 | Normal | 1
#12 Peak Hip Extension in Stance | 0 | Normal | 1
#13 Peak Hip Flexion during Swing | 0 | Normal | 1
#15 Pelvic Rotation at Mid-Stance | 0 | Normal | 1
Explanation of Abnormal Items:
#1 Initial Contact → 2 score (Toe contact)
#7 Max Ankle Dorsiflexion in Swing → 1 score (Increased dorsiflexion)
#10 Knee Position in Terminal Swing → 1 score (Moderate flexion)
#5 Foot Rotation → 1 score (Moderate excessive external rotation)
#17 Maximum Lateral Displacement → 1 score (Moderate excessive right tilt)

E.

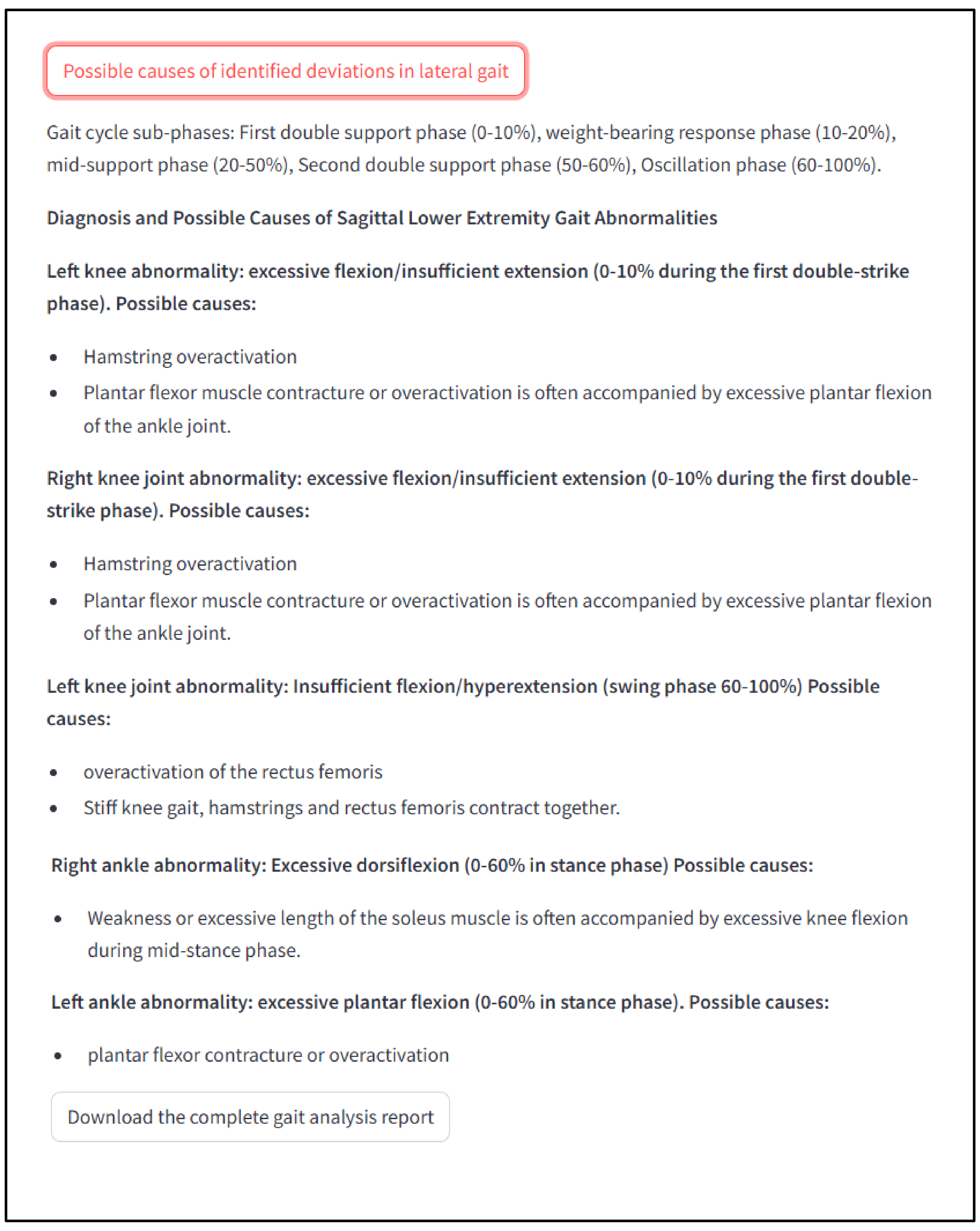
Possible causes of identified deviations in lateral gait
Gait cycle sub-phases: First double support phase (0-10%), weight-bearing response phase (10-20%), mid-support phase (20-50%), Second double support phase (50-60%), Oscillation phase (60-100%).
Diagnosis and Possible Causes of Sagittal Lower Extremity Gait Abnormalities
Left knee abnormality: excessive flexion/insufficient extension (0-10% during the first double-strike phase). Possible causes:
• Hamstring overactivation
• Plantar flexor muscle contracture or overactivation is often accompanied by excessive plantar flexion of the ankle joint.
Right knee joint abnormality: excessive flexion/insufficient extension (0-10% during the first double-strike phase). Possible causes:
• Hamstring overactivation
• Plantar flexor muscle contracture or overactivation is often accompanied by excessive plantar flexion of the ankle joint.
Left knee joint abnormality: Insufficient flexion/hyperextension (swing phase 60-100%) Possible causes:
• overactivation of the rectus femoris
• Stiff knee gait, hamstrings and rectus femoris contract together.
Right ankle abnormality: Excessive dorsiflexion (0-60% in stance phase) Possible causes:
• Weakness or excessive length of the soleus muscle is often accompanied by excessive knee flexion during mid-stance phase.
Left ankle abnormality: excessive plantar flexion (0-60% in stance phase). Possible causes:
• plantar flexor contracture or overactivation
Download the complete gait analysis report

F.
