# Supplementary material for: Co-Designing a Clinician-Centered Video Gait Assessment System for Cerebral Palsy Based on the Framework for Co-Design of Clinical Practice Tools: Iterative Development and Formative Evaluation Study
Source: J Med Internet Res. 2026 Aug 21;28:e92823. doi: 10.2196/92823 (PMC13496319; doi:10.2196/92823)
Supplement: Supplementary file 2 [file jmir-v28-e92823-s001.docx]

**Multimedia Appendix 1. System Usability Scale questionnaire and semistructured interview guide used to follow the think-aloud exercises**

**System Usability Scale (SUS) questionnaire**

**Purpose:**

To assess participants perceived usability of the video-based gait analysis (VGA) system following completion of the think-aloud tasks. Each statement below was rated on a 5-point Likert scale ranging from 1 = Strongly disagree to 5 = Strongly agree.

| **No.** | **Item** | **Response scale** |
| --- | --- | --- |
| 1 | I think that I would like to use this system frequently. | 1 2 3 4 5 |
| 2 | I found the system unnecessarily complex. *(R)* | 1 2 3 4 5 |
| 3 | I thought the system was easy to use. | 1 2 3 4 5 |
| 4 | I think that I would need the support of a technical person to be able to use this system. *(R)* | 1 2 3 4 5 |
| 5 | I found the various functions in this system were well integrated. | 1 2 3 4 5 |
| 6 | I thought there was too much inconsistency in this system. *(R)* | 1 2 3 4 5 |
| 7 | I would imagine that most people would learn to use this system very quickly. | 1 2 3 4 5 |
| 8 | I found the system very cumbersome to use. *(R)* | 1 2 3 4 5 |
| 9 | I felt very confident using the system. | 1 2 3 4 5 |
| 10 | I needed to learn a lot of things before I could get going with this system. *(R)* | 1 2 3 4 5 |

**Scoring:**

For odd-numbered items, subtract 1 from the participant’s score.

For even-numbered (reverse-scored) items, subtract the score from 5.

Sum the adjusted scores and multiply the total by 2.5 to obtain the overall SUS score (range 0–100).

Higher scores indicate greater perceived usability.

**Semi-structured interview guide used to follow the think-aloud exercises**

1) General impressions
   a) Did you find it easy or difficult to complete the assigned tasks using the system?
   b) Were there any points where you felt uncertain about what to do next?
   c) Did you find the overall layout and navigation intuitive?

2) Specific interface and functionality feedback
   a) What aspects of the interface helped you most when analysing gait or reviewing the report?
   b) Were there features or labels that were confusing or unclear?
   c) Did the visual feedback or error messages appear at the right time and in a useful way?
   d) How did you find the readability (font size, colour contrast, report layout)?

3) Clinical relevance and workflow integration
   a) Do you think the information presented in the system/report would be useful in your clinical practice?
   b) Are there additional data or visualisations that would make the report more clinically meaningful?
   c) At what point in your clinical workflow do you think this system could be used?
   d) What kinds of clinical applications do you think it would be most helpful for?

4) Improvement and future use
   a) What features would you like to see added or removed in future versions?
   b) Would you consider using this system in your daily work if it were available online? Why?
