# Supplementary material for: Co-Designing a Clinician-Centered Video Gait Assessment System for Cerebral Palsy Based on the Framework for Co-Design of Clinical Practice Tools: Iterative Development and Formative Evaluation Study
Source: J Med Internet Res. 2026 Aug 21;28:e92823. doi: 10.2196/92823 (PMC13496319; doi:10.2196/92823)
Supplement: Supplementary file 3 [file jmir-v28-e92823-s002.docx]

**Multimedia Appendix 2. Examples of topic guide questions used in the postsimulation focus group**

**Examples of topic guide questions used in the post-simulation focus group**

Did you encounter any difficulties or confusion when using the prototype? If so, please describe. *(prompts: workflow steps, navigation, terminology, clarity of visual elements, report layout)*

Compared with your usual practice, how did the prototype affect your ability to understand or communicate the patient’s gait problems?

What impact did the automatically generated report have on your communication with colleagues or your clinical decision-making? *(positive / neutral / negative?)*

Thinking about what it might be like to use the tool in routine practice:

*• How well would this tool fit with your existing assessment workflow?*

*• How might the tool help or hinder clinical decision-making or patient discussions?*

What changes might improve the design or content of the tool? *(prompts: workflow alignment, terminology, visual hierarchy, clarity of deviation labels; in an ideal world?)*

Did you identify any potential risks when using the tool? *(prompts: over-reliance on automated outputs, misinterpretation, disruptions during patient handling)*

What forms of training or support would help you feel confident using this system in routine clinical practice?
