# Supplementary material for: Co-Designing a Clinician-Centered Video Gait Assessment System for Cerebral Palsy Based on the Framework for Co-Design of Clinical Practice Tools: Iterative Development and Formative Evaluation Study
Source: J Med Internet Res. 2026 Aug 21;28:e92823. doi: 10.2196/92823 (PMC13496319; doi:10.2196/92823)
Supplement: Supplementary file 4 [file jmir-v28-e92823-s003.docx]

**Multimedia Appendix 3. Context-of-use analysis and design refinements for the system**

Key components of context-of-use analysis for the video-based gait analysis (VGA) system.

| Component | Explanation |
| --- | --- |
| Clinician-oriented design | Developed for rehabilitation professionals, including physiotherapists, orthopaedists, and gait analysts, who have varying levels of technical experience. The interface was therefore designed to minimise operational barriers, use clear clinical terminology, and require minimal manual configuration to support efficient use in routine practice. |
| Usable across routine clinical settings | Designed for use in outpatient clinics and gait laboratories equipped with standard lighting and sufficient walking space, without the need for specialised motion capture systems or controlled environments. |
| Adaptable to different patient presentations | Configured to accommodate diverse gait patterns among children with cerebral palsy, with both automatic and manual segmentation options. |
| Simple and time-efficient to use | Required minimal setup and offered straightforward video upload, automatic processing, and one-click report generation to support use within time-limited consultations. |
| Compatible with existing documentation | Generated outputs in a standardised PDF format that could be integrated into existing clinical documentation systems or patient records. |
| Technically lightweight | Operable on general-purpose computers, using a single RGB camera, reducing hardware costs and enabling deployment in low-resource settings. |

Examples of design refinements from Design 1 to Design 2.

| Design aspect | Design 1 | Design 2 | Usability principles applied |
| --- | --- | --- | --- |
| Visual hierarchy and readability | Inconsistent font sizes and colours; dense layout | Unified typography and colour scheme; simplified layout for clearer visual grouping | Aesthetic and minimalist design |
| Error handling | No feedback for invalid file uploads or processing errors. | Added real-time error messages with retry prompts and guidance for correcting the issue. | Error prevention |
| Operation feedback | No confirmation after successful operations | Added real-time success messages confirming task completion (e.g., “Processing completed successfully”) | Visibility of system status |
| Accessibility and deployment | Local desktop prototype | Added online web-based version accessible via URL | Flexibility and efficiency of use |
