# Supplementary material for: Co-Designing a Clinician-Centered Video Gait Assessment System for Cerebral Palsy Based on the Framework for Co-Design of Clinical Practice Tools: Iterative Development and Formative Evaluation Study
Source: J Med Internet Res. 2026 Aug 21;28:e92823. doi: 10.2196/92823 (PMC13496319; doi:10.2196/92823)
Supplement: Supplementary file 6 [file jmir-v28-e92823-s006.docx]

**Multimedia Appendix 6. Implementation guideline of the gait analysis system**

*The gait analysis system was developed primarily for use in China, and the original implementation guideline was written in Chinese. The English version included in this supplement is provided for reference only and represents a translation of the original document. It has not been clinically or linguistically standardised for use in English-speaking clinical contexts.*

**1. Product Overview**

The Online Gait Analysis System for Children with Cerebral Palsy is a computer-vision–based assessment platform designed specifically for children with CP. By combining image-based pose estimation and automated scoring algorithms, the system provides clinicians and rehabilitation therapists with a convenient, objective, and quantitative tool for gait evaluation.

Using a standard smartphone camera, the system captures gait videos, automatically detects key body landmarks, and generates evaluation results based on the Edinburgh Visual Gait Score (EVGS). The system supports the full workflow of video upload, keypoint extraction, deviation detection, and score generation, greatly reducing reliance on subjective clinical judgement.

Its cloud-based deployment allows gait assessment to be performed at home, in community clinics, or through tele-rehabilitation platforms, thereby improving accessibility. The system aims to enable continuous and trackable gait assessment for children with CP and to support clinicians in developing personalised rehabilitation strategies and timely interventions.

**2. Gait Video Acquisition Instructions**

**2.1 Target Users**

Children with cerebral palsy who can walk independently without assistive devices.

**2.2 Equipment Requirements**

- Smartphone
- A straight walkway at least 4 meters long
- Tripod or phone stabiliser
- Ensure video quality ≥1080p and frame rate ≥60 FPS

**2.3 Camera Placement**

**1) Tripod Setup**

The smartphone should be mounted on a tripod, with the camera lens positioned at approximately hip height (0.5–0.8 m from the ground).

**2) Side-View Recording**

- Phone placed horizontally
- Positioned on one side of the walkway
- Camera perpendicular to the ground
- Rear camera facing the walking path
- Distance from walkway center: ~3.5 m
- Ensure the full body of the child and the entire walkway length remain visible throughout the video

**3) Front-View Recording**

- Phone placed vertically
- Rear camera facing the walkway
- Child walks toward the camera from far to near
- Child should remain centered and facing the camera at all times

**2.4 Testing Procedure**

- Participants walk barefoot and wear fitted clothing (e.g., tight shorts and a contrasting top)
- Warm-up walking is recommended to familiarise the child with the environment
- Record three valid side-view videos (left to right walking) and three valid front-view videos (walking toward the camera)
- Each video must:
  - Clearly display the child’s full body
  - Maintain consistent image quality
  - Capture **at least three gait cycles per limb**

**3. System Operation Instructions**

**3.1 Accessing the System**

Open the platform at: [**https://8.138.167.96/gait/**](https://8.138.167.96/gait/)
Enter basic patient information (name, height, etc.)

**3.2 Side-View Video Processing**

1. **Upload the recorded side-view video**
   (Drag-and-drop or select via “Browse files”)
2. **Start posture estimation** by clicking “Start Posture Assessment”
3. **Stop posture estimation**
   A green message “Posture recognition completed” will appear upon success

4. **Compute joint angles**
   Click “Compute Joint Angles”
   A green message confirms completion

5. **Extract gait cycles**
   - The system automatically identifies key gait events
   - Verify accuracy:
     - Peaks represent foot-strike
     - Troughs represent toe-off
     - Ensure first-to-last peak/trough detection sequence is correct

**Manual correction (if needed)**

- Scroll through the video and confirm foot-strike and toe-off frames
- Enter the correct frame numbers
- Requirements:
  - Gait cycle must begin at foot-strike
  - Events must follow proper sequential order
  - Each foot’s strike and toe-off must be marked
  - Users may define how many cycles to include—no need to analyse all cycles

Click **“Save Corrections”** when event editing is completed

6. **Spatiotemporal parameter calculation**
   - Click “Compute Spatiotemporal Parameters”
7. **Kinematic analysis**
   - Click “Kinematic Analysis” to view joint angle time-series plots

**3.3 Front-View Video Processing**

1. Upload the front-view video and ensure the child remains centered
2. Click **“Start Front-View Assessment”**
3. Click **“Stop Front-View Assessment”**

**3.4 EVGS Scoring**

Click “Compute EVGS”
The Edinburgh Visual Gait Score results will be displayed.

**3.5 Sagittal Plane Deviation and Cause Identification**

Click “Sagittal Gait Deviations and Possible Causes”
The system outputs deviation types and likely biomechanical causes.

**3.6 Report Download**

Click **“Download Full Gait Analysis Report”**
A complete PDF report will be generated.

**4. Error Handling**

**4.1 Gait Cycle Extraction Errors**

Most errors occur due to inaccurate key event detection. Possible issues include:

- Missing peak (foot-strike) detection
- Incorrect trough (toe-off) identification
- Incorrect classification of movement phases

**Suggested solutions:**

- Manually adjust the key events
- Re-upload higher-quality video
- Trim the original video and retry

**4.2 EVGS Scoring Errors**

Usually caused by inaccurate **mid-stance** event identification.

**Solutions:**

- Re-check key gait events
- Re-upload alternative videos

**5. User Training**

Short training sessions are recommended for new users, covering:

- Proper video capture technique (camera angle, distance, visibility)
- Manual correction of gait events when needed
- Interpreting spatiotemporal and kinematic results
- Understanding EVGS scoring and automated explanations
- Exporting and integrating reports into clinical workflow

Training can be delivered through short instructional videos or a written quick-start guide.
