# Supplementary material for: Co-Designing a Clinician-Centered Video Gait Assessment System for Cerebral Palsy Based on the Framework for Co-Design of Clinical Practice Tools: Iterative Development and Formative Evaluation Study
Source: J Med Internet Res. 2026 Aug 21;28:e92823. doi: 10.2196/92823 (PMC13496319; doi:10.2196/92823)
Supplement: Supplementary file 7 [file jmir-v28-e92823-s004.docx]

**Multimedia Appendix 4. Examples of the initial draft of the prototype (design 2)**

C.

D.

E.
